# Reduction of extracellular vimentin in blood provides protection against SARS-CoV-2 infection

**DOI:** 10.1080/21505594.2025.2568052

**Published:** 2025-10-07

**Authors:** Hae-Mi Kim, Mingda Wang, Chongkai Zhai, Sura Kim, Jungha Park, Seong-Tshool Hong

**Affiliations:** a Department of Biomedical Sciences and Institute for Medical Sciences, Jeonbuk National University Medical School, Jeonju, South Korea; b Department of Critical Care Medicine, Shandong Provincial Hospital Affiliated to Shandong First Medical University, Jinan, China; c Animal Diseases and Public Health Engineering Research Centre of Henan Province, Luoyang Polytechnic, Luoyang, China

**Keywords:** eVIM, COVID-19, hzVSF-v13, Roborovski SH101 hamsters

## Abstract

*In vitro* studies have repeatedly showed that extracellular vimentin (eVIM) promotes the penetration of viruses by acting as an adhesion factor, suggesting that reducing eVIM density in the blood could be an effective approach to treat viral infections. However, despite solid evidence, it has not been previously investigated whether eVIM plays a pathogenic role during viral infections *in vivo* experiments. Here, we provide *in vivo* evidence that eVIM plays a critical role during viral infections. The severity of COVID-19 in Roborovski SH101 hamsters was positively correlated with blood concentrations of eVIM during the infection period. The reduction of blood eVIM in the SARS-CoV-2-infected Roborovski SH101 hamster through intravenous injection of hzVSF-v13, a humanized anti-eVIM monoclonal antibody, dramatically improved disease manifestations, such as body weight reduction, body temperature, death rate, and more. It also inhibited the formation of blood clots and systemic inflammation compared to remdesivir or a SARS-CoV-2 neutralizing antibody (6D11F2). Histological examination confirmed the significantly better therapeutic efficacy of anti-eVIM compared to remdesivir or the neutralizing antibody. Quantification of SARS-CoV-2 in the hamsters’ lungs revealed that the viral titer in the anti-eVIM-treated groups was 77% to 80% lower than that in the infection group, while remdesivir and the neutralizing antibody resulted in reductions of 41.9% and 34.7% to 44.1%, respectively. We believe that this work provides a foundation for the development of hzVSF-v13 as an antiviral drug for COVID-19.

## Introduction

The COVID-19 pandemic caused by SARS-CoV-2 has recently emerged as a new infectious disease, posing a serious global threat to public health and the economy. The abrupt emergence of SARS-CoV-2 is one of the most significant scientific questions of recent times. Genome analyses have revealed that the genomes of many zoonotic beta-coronaviruses in bats and pangolins share homology with the genome of SARS-CoV-2 [1–5]. However, none of these zoonotic virus genomes exhibit more than 98% similarity to that of SARS-CoV-2 [6–8], suggesting that these SARS-CoV-2-related coronaviruses are not direct ancestor of SARS-CoV-2. Despite disappointment in the search for the origin of SARS-CoV-2 through sequence comparison analyses, a recent approach – the whole prototype proteome sequence analysis – indicated that SARS-CoV-2 emerged through an unusual replacement of a motif in the spike protein of SARS-CoV with the motif in a membrane protein of *P. malariae* [9].

The impact of SARS-CoV-2 on public health and society has been as unique as its emergence. SARS-CoV-2 has spread rapidly across the globe with generating a diverse group of variants [10–12]. The recent development of prophylactic vaccines, therapeutic agents, and natural herd immunity have partially mitigated the spread of SARS-CoV-2 [13,14]. However, the constant and rapid emergence of SARS-CoV-2 variants, exemplified by omicron and its subsequent variants [15,16], raises questionable about the possibility of eradicating the virus from society. This necessitates ongoing efforts to develop new therapeutic drugs for COVID-19. Therefore, the significance of scientific research on new drug target for COVID-19 cannot be overstated. In this regard, vimentin should be actively explored as a potential drug target of viral infections, including COVID-19, due to its unique role in the pathogenesis of viral infections.

Vimentin is a type III intermediate filament protein that plays a vital role in maintaining the structural integrity and organization of the cytoplasm in mesenchymal cells, primarily by providing mechanical support and aiding in organelle positioning [17,18]. While vimentin is traditionally characterized as an intracellular protein, it also exists in extracellular form as a circulating molecule in blood, known as extracellular vimentin (eVIM) [19]. Intracellular vimentin is well recognized for its contribution to overall cell architecture and resilience; however, emerging evidence suggests that eVIM is particularly important in the context of viral infections [20–22]. In this extracellular capacity, eVIM has been shown to serve as an attachment factor or coreceptor for several viruses – among them SARS-CoV [23], cowpea mosaic virus [24], Japanese encephalitis virus [25], dengue virus [26], and human papillomavirus [27]—thereby influencing viral entry and pathogenesis. Recently, *in vitro* experiment has indicated that eVIM binds to SARS-CoV-2 S-protein, facilitating SARS-CoV-2 entry into human endothelial cells and suggesting a significant role in the pathogenesis of severe COVID-19 [28]. These research findings collectively highlight that eVIM interacts with viral receptors on host cells to facilitate viral entry. It functions as a co-factor, bridging the virus and its primary receptor on the host cell surface. The cooperative role of eVIM with viral receptors enhances viral attachment, fusion, and entry. Consequently, the removal of eVIM inhibits infectivity, while the addition of eVIM enhances it [27,28].

However, despite strong *in vitro* evidence, it has not yet been investigated whether reducing eVIM concentration in the blood actually inhibits viral infectivity in *in vivo* experiments. Here, we demonstrate that the reduction of eVIM using an anti-eVIM monoclonal antibody, hzVSF-v13, confers resistance to SARS-CoV-2 infection *in vivo*, indicating that eVIM plays a critical pathogenic role during viral infections. Overall, the therapeutic efficacy of hzVSF-v13 for COVID-19 was superior to that of Remdesivir or a SARS-CoV-2 neutralizing antibody.

## Methods

### Ethics statement

This study was approved by the Animal Use and Care Committee of the Jeonbuk National University (permit number: JBNU 2021–0184, NON2022-102) conformed to the Guidelines for the Care and Use of Laboratory Animals based on the ARRIVE [29]. All methods were performed in accordance with the relevant guidelines and regulations. All the animals used in this research were managed in a manner consistent with CDC/ABSA/WHO guidelines for the prevention of human infection with the SARS- CoV-2 virus.

### Viruses, cells and drugs

The SARS-CoV-2 strain HB-01 was obtained from the National Culture Collection for Pathogens (NCCP, Korea) of the Korea Disease Control and Prevention Agency (KDCA). The complete genome sequence can be found at either GISAID (identifier: BetaCoV/Wuhan/IVDC-HB-01/2020|EPI_ISL_402119) or the China National Microbiological Data Center (accession number NMDC10013001 and genome accession number MDC60013002-01). Preparation of seed SARS-CoV-2 stocks and isolation of the virus were conducted using Vero cells, as described previously [30], which were maintained in Dulbecco’s modified Eagle’s medium (DMEM; Gibco, USA) supplemented with 10% fetal bovine serum (FBS; Gibco, USA), 100 IU/mL penicillin (Gibco), and 100 μg/mL streptomycin (Gibco), and incubated at 37°C, 5% CO_2_. The hzVSF-v13, a humanized IgG4 monoclonal antibody which strongly binds to eVIM [31], was provided by ImmuneMed (Chuncheon, South Korea). The remdesivir was purchased by MedKoo Biosciences (329511, USA). The anti-SARS-CoV-2 neutralizing monoclonal antibody was purchased by GenScript (6D11F2, USA).

### Animal experiments

The Roborovski hamster (*Phodopus roborovskii*) strain SH101 was obtained from Alpha Biochemicals Co. (http://alphabiochemicals.com). Experimental hamsters of appropriate age were selected, and groups of hamsters were placed in different cages. For animal, food (10% kcal as fat intake; D12450B; Research Diets Inc.) and water were provided. A 12-h light–dark cycle was maintained in a room of the ABL3 lab of Jeonbuk National University with controlled temperature (22°C ±1°C) and humidity (55% ± 5%) for 2 weeks. After 2 weeks of acclimation in the ABL3 lab, the virus infection and drug injection according to the groups proceeded as follows. To ensure effective bioavailability and therapeutic efficacy, hzVSF-v13 was administered via *iv* injection at high dose of 30 mg/kg and low dose of 10 mg/kg, as determined based on our preliminary dose–response studies and prior reports [32]. Virus infection was performed after anesthesia. (1) The un-infection control group: 50 μL of DMEM was injected into the Roborovski SH101 hamster’s nasal instead of SARS-CoV-2, and 100 μL of saline was intravenously (*i.v*.) injected twice at 0 and 2 dpi (day post infection). (2) The antibody control group: 50 μL of DMEM was injected into the Roborovski SH101 hamster’s nasal instead of SARS-CoV-2, and hzVSF-v13 at 10 mg/kg was *i.v*. injected twice at 0 and 2 dpi. (3) In the infection control group: 50 μL of SARS-CoV-2 solution (10^5^ TCID_50_) was infected intranasally, and 100 μL of saline was *i.v*. injected twice at 0 and 2 dpi. (4) For hzVSF-v13 efficacy evaluation: 50 μL of SARS-CoV-2 solution (10^5^ TCID_50_) was infected intranasally, and hzVSF-v13 was injected twice (0 and 2 dpi) into the Roborovski SH101 hamster at concentrations of 10 and 30 mg/kg, respectively. (5) The Remdesivir treatment group: 50 μL of SARS-CoV-2 solution (10^5^ TCID_50_) was infected intranasally, and remdesivir was *i.v*. injected at a dose of 5 mg/kg on day 0 and 2.5 mg/kg per day from 1 to 3 dpi. (6) The SARS-CoV-2 neutralizing monoclonal antibody (GenScript, 6D11F2) treatment groups: 50 μL of SARS-CoV-2 solution (10^5^ TCID_50_) was infected intranasally, and the SARS-CoV-2 Neutralizing Monoclonal Antibody was injected twice (0 and 2 dpi) into the Roborovski SH101 hamster at concentrations of 10 and 30 mg/kg, respectively. For a total of 8 groups, 18 hamsters each group were used in the *in vitro* study. The hamsters’ health status was checked daily for 9 d. In the same manner as described previously [30] for human SARS-CoV-2 infection, the time at which hamsters reached severe infection levels was used as a criterion. Therefore, 2 dpi before indicating the severe infection level, 4 dpi indicating the severe infection level, and 8 dpi expected to be recovered by the drug were used. At 2 dpi, 4 dpi, and 8 dpi, randomly selected hamsters from each group were anesthetized with isoflurane and sacrificed after 10 min.

### Body temperature and weight measurement

The body temperatures of the Roborovski SH101 hamsters were measured by a high-precision thermal photographic method [33,34]. The entire bodies of the hamsters were photographed with an FLIR thermal imaging camera, and the thermal images were analyzed by DirA program (FLIR Tools) to measure the body temperature. The highest temperature spot on the chest, near the location of the lung, was identified in each Roborovski SH101 hamster to determine the body temperature. The body weight of the Roborovski SH101 hamsters were measured, and the weight change rate (%) was calculated as [current weight/0 dpi weight × 100]. All data are presented as the mean ± standard deviation and were compared using paired Student’s t-test.

### Blood profile analysis

The hypercoagulable state of the SARS-CoV-2-infectd Roborovski SH101 hamsters was analyzed by D-dimer and fibrin degradation products (FDP). Whole blood was collected in a 1.5 mL Eppendorf micro-centrifuged tube by cardiac puncture. Blood serum was separated by centrifugation at 2,500 × g for 10 min and stored at 25°C until used. The D-dimer and FDP concentration of serum samples were determined by Hamster D-dimer (D2D) ELISA Kit (MBS012417, MyBioSource), Hamster Fibrinogen Degradation Product (FDP) ELISA Kit (MBS005821, MyBioSource).

Total vimentin was quantified using and ELISA kit (7789, Cell Signaling). The amount of vimentin that does not bind to hzVSF-v13 was measured by treating an anti-Human IgG Magnetic Beads (801–101, RayBiotech). One hundred μL of the beads was washed by 1 mL of TBST buffer twice and resuspended in 450 μL of TBST buffer. Fifty microliter of serum was added to the beads. After gentle mixing for 30 min, they were magnetically separated until the supernatant became clear. Taken supernatant was collected, and the amount of vimentin that did not bind to hzVSF-v13 was measured using the ELISA kit.

The concentrations of IL-1β, IL-6, TNF-α and IL-8 in serum samples were quantified using the Hamster IL-1β (interleukin-1β) ELISA Kit (MBS7612363, MyBioSource), Hamster IL-6 (interleukin-6) ELISA Kit (MBS7606648, MyBioSource), Hamster TNF-α (Tumor Necrosis Factor-α) ELISA Kit (MBS7606475, MyBioSource), Hamster IL-8 (interleukin-8) ELISA Kit (MBS1604081, MyBioSource). All ELISA kits were conducted according to the manufacturer’s protocol and measured with a SpectraMax Plus 384 plate reader (Molecular Devices).

### RNA isolation and quantitation of SARS-CoV-2 titters

RNA isolation and quantitation assay has been previously described in detail [30]. Total RNA was extracted from the supernatants of the organ homogenates using the RNeasy Mini Kit (QIAGEN), and reverse transcription was performed using the PrimerScript RT Reagent Kit (TaKaRa) following the manufacturers’ instructions. RT – qPCR reactions were performed using the PowerUp SYBG Green Master Mix Kit (Applied Biosystems, Waltham). Samples were processed in duplicate using the following cycling protocol: 50°C for 2 min, 95°C for 2 min, followed by 40 cycles at 95°C for 15 s and 60°C for 30 s, and then 95°C for 15 s, 60°C for 1 min, 95°C for 45 s. The primer sequences used for RT – qPCR is targeted against the envelope (E) gene of SARS-CoV-2 and are as follows: forward: 5′-GCCTCTTCTCGTTCCTCATCAC −3′, reverse: 5′- AGCAGCATCACCGCCATTG −3′. PCR products were verified by sequencing using the dideoxy method on an ABI 3730 DNA sequencer (Applied Biosystems, Waltham, MA, USA). The obtained sequencing reads were compared with the reads from the NCBI database. The SYBR green real-time PCR standard curve was generated by serial ten-fold dilutions of recombinant plasmid with a known copy number (from 7 × 10^7^ to 7 × 10^1^ copies per μL). These dilutions were evaluated and used as quantification standards to construct the standard curve by plotting the plasmid copy number against the corresponding threshold cycle values (Ct). Results were expressed as log10-transformed numbers of genome equivalent copies per ml of sample. The Ct values of each sample were used to quantitate the virus titers by using the standard curve.

### H&E, MT and PAS staining

Lungs were isolated from Roborovski SH101 hamster at 2 and 4 d post-infection (dpi) respectively. Immediately after isolation, the lungs were fixed using 10% neutral-buffered formalin, embedded in paraffin, and sectioned to be placed on glass microscopic slides (5 μm thickness). The 5 μm sections were then stained with hematoxylin and eosin (H&E), Masson Trichrome (MT) and Periodic acid-Schiff (PAS) stains. The stained tissue images were observed using an Apero ScanScope FL microscope (Leica Biosystems, Germany). The overall severity of each lung section is expressed the mean of the scores of the observed microscopic fields. Lung fibrosis was quantified in histological specimens using a numerical scale. The severity of fibrotic changes in each observed microscopic field of a given lung section was assessed and scored using a modified Ashcroft scale from 0 to 8 [35].

### Statistical analysis

The statistical differences between groups were calculated with one-way ANOVA multiple comparisons test. Statistical analyses and figure preparation were performed using GraphPad Prism (version 5). Tests and calculated p-values are indicated on the figures. Differences with *p* ≤ 0.05 are considered significant.

## Results

### The severities of COVID-19 in Rovoroski SH101 hamster were positively corelated with the concentrations of eVIM

To investigate the *in vivo* pathogenic role of eVIM during viral infection, we initially assessed eVIM concentrations of Roborovski SH101 hamster post-infection with SARS-CoV-2, which serves as an ideal model for studying SARS-CoV-2 viral infection [30]. Nasal inoculation of the hamsters with SARS-CoV-2 led to the development of typical clinical symptoms resembling COVID-19, including snuffling, labored breathing, dyspnea, cough, hunched posture, progressive weight loss, ruffled fur, and high fever accompanied by shaking chills (Supplemental Table S1). Histological examinations also showed a right-predominated pneumonia, confirming COVID-19 (Figure S1-S2).

**Figure 1.**
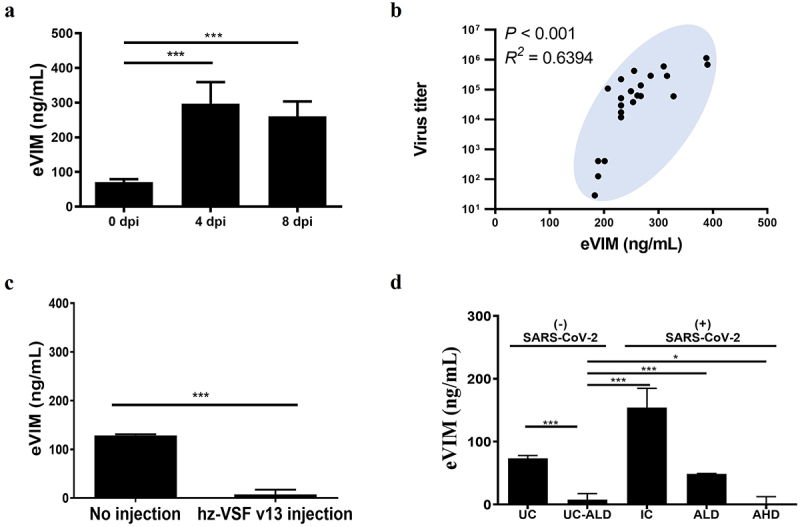
The severity of SARS-CoV-2 infection in Roborovski SH101 correlates with eVIM, and anti-eVIM reduces the blood concentration of eVIM. (a) eVIM concentrations in the serum of Roborovski SH101 hamsters at 0 dpi (days post infection) (*n* = 5), 4 dpi (*n* = 9) and 8 dpi (*n* = 10) of SARS-CoV-2. (b) The correlation analysis between the SARS-CoV-2 viral titer of the infected lung and eVIM concentrations (*n* = 22) (*R*^*2*^ = 0.6394, *p* < 0.001). (c) The blood eVIM concentration of Roborovski SH101 hamsters without and with *i.V*. injection (4 dpi, *n* = 6) of anti-eVIM (hzVSF-v13 10 mg/kg). The eVIM concentrations were measured after reaction with magnetic beads coated with goat anti-human IgG to remove the hzVSF-v13-eVIM complex. (d) The blood eVIM concentration of Roborovski SH101 hamsters infected with SARS-CoV-2 or uninfected control (*n* = 6). The eVIM concentrations were measured after reaction with magnetic beads coated with goat anti-human IgG to remove the hzVSF-v13-eVIM complex. UC, uninfection control; UC-ALD, uninfected hamster but injected with 10 mg/kg of hzVSF-v13; IC, infection control (the SARS-CoV-2-infected hamsters); ALD, the SARS-CoV-2-infected hamsters treated with low dose of anti-eVIM (hzVSF-v13 10 mg/kg); AHD, the SARS-CoV-2-infected hamsters treated with high dose of anti-eVIM (hzVSF-v13 30 mg/kg. The data are presented as mean ± SD. The statistical significances are marked on the graphs as *p < 0.05, **p < 0.01 and ***p < 0.001.

Concomitant with the development of COVID-19, Roborovski SH101 hamsters infected with SARS-CoV-2 increased eVIM concentrations by 420% at 4 d post-infection (dpi), as demonstrated in Figure 1(a). Notably, the elevated levels of eVIM in the blood remained consistently high throughout the terminal stages of COVID-19 progression, suggesting a crucial pathological role for eVIM during SARS-CoV-2 infection. Further statistical analysis substantiated the correlation between SARS-CoV-2 infection and the induction of eVIM. As illustrated in Figure 1(b), the correlation analysis revealed a significant relationship, with the actual viral titer in the infected lung showing a positive correlation with eVIM concentrations (R^2^ = 0.6394, *p* < 0.001). This positive correlation between the SARS-CoV-2 viral titer and eVIM concentrations underscored that the severity of COVID-19 in Roborovski SH101 hamsters was indeed positively associated with eVIM concentrations.

### The humanized anti-vimentin monoclonal antibody, hzVSF-v13, efficiently bound to eVIM in the serum of the Roborovski SH101 hamster

The hzVSF-v13 antibody is a humanized IgG4 monoclonal antibody which strongly binds to eVIM [30]. To evaluate how hzVSF-v13 affects circulating eVIM levels, sera were collected from Roborovski SH101 hamsters that had received an *i.v*. injection of hzVSF-v13 as well as from untreated control hamsters. These sera were then incubated with magnetic beads coated with goat anti-human IgG, which selectively bind to human IgG molecules. Because hzVSF-v13 strongly binds eVIM, any eVIM – hzVSF-v13 complexes present in the serum were also captured by the magnetic beads. Upon centrifugation, these bead-bound complexes were removed from the serum, leading to a measurable reduction in the concentration of free eVIM (Figure 1(c)). This demonstrates that hzVSF-v13 effectively neutralizes eVIM by forming complexes that can be readily isolated from the bloodstream, thereby lowering the overall level of circulating eVIM.

Having confirmed the neutralization effect of hzVSF-v13 on eVIM in the serum, we proceeded to validate whether hzVSF-v13 could reduce serum eVIM concentrations in SARS-CoV-2-infected hamsters. As shown in Figure 1(d), addition of hzVSF-v13 into the collected sera of hamsters, both with and without SARS-CoV-2 infection led to a significant reduction in blood eVIM concentrations. This outcome clearly indicated that hzVSF-v13 bound to eVIM in the serum of Roborovski SH101 hamsters, suggesting that the interaction between hzVSF-v13 and eVIM, following intravenous treatment, could interfere with the pathogenic role of eVIM in viral infection.

### IV. treatment of COVID-19 Roborovski SH101 hamsters with hzVSF-13 dramatically improved the disease manifestations

The effect of hzVSF-v13 on eVIM prompted us to evaluate the therapeutic potential of hzVSF-v13 in SARS-CoV-2-infected Roborovski SH101 hamsters after *i.v*. treatment. The hamsters were infected with SARS-CoV-2 and then *i.v*. injected twice, at 0 and 2 d dpi, with different doses of hzVSF-v13 (anti-eVIM low dose, ALD, 10 mg/kg, and anti-eVIM high dose, AHD, 30 mg/kg), an anti-SARS-CoV-2 neutralizing monoclonal antibody (GenScript, 6D11F2) (neutralizing antibody low dose, NLD, 10 mg/kg, and neutralizing antibody high dose, NHD, 30 mg/kg), or the therapeutic dose of remdesivir (REM, 5 mg/kg) (*n* = 6 for each group). We assessed various disease manifestations in the hamsters, including weight loss, appearance, activity, labored breathing, dyspnea, cough, hunched posture, ruffled fur, shaking chills, and body temperature. Other than natural death, the hamsters were euthanized and classified as a dead individual if their body weight decreased to more than 20% with hypothermia, indicating morbidity or death.

**Figure 2.**
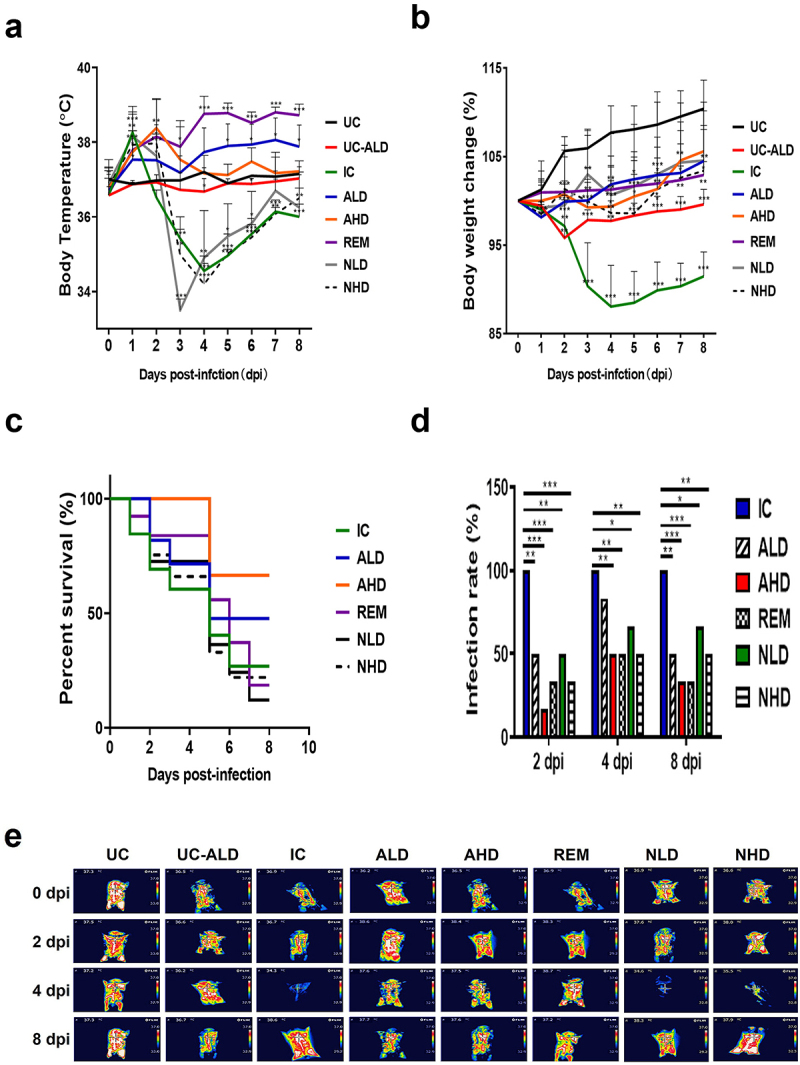
Anti-eVIM improves the disease manifestations of COVID-19 Roborovski SH101 hamsters. (a) The body temperature changes on the chests of each Roborovski SH101 hamster group. The body temperatures were measured by selecting the highest temperature spot on the thermal images and are presented as mean ± SD. (b) The body weight changes of each Roborovski SH101 hamster group during the experimental period. (c) The survival rates of each Roborovski SH101 hamster group. The survival rates were recorded daily for 9 d. (d) The infection rates in the lung of the SARS-CoV-2 infected Roborovski SH101 hamster groups. The infection rates were counted from the lung images and pathological examination results of the infected Rovorovski S101 hamsters at 2, 4 and 8 dpi. (e) Their representative infrared thermographic images. UC, uninfection control; UC-ALD, uninfected hamster but injected with 10 mg/kg of hzVSF-v13; IC, infection control (the SARS-CoV-2-infected hamsters); ALD, the SARS-CoV-2-infected hamsters treated with low dose of anti-eVIM (hzVSF-v13 10 mg/kg); AHD, the SARS-CoV-2-infected hamsters treated with high dose of anti-eVIM (hzVSF-v13 30 mg/kg); REM, the SARS-CoV-2-infected hamsters treated with the therapeutic dose of remdesivir (5 mg/kg); NLD, the SARS-CoV-2-infected hamsters treated with the anti-SARS-CoV-2 neutralizing monoclonal antibody (GenScript, 6D11F2) (10 mg/kg); NHD, the SARS-CoV-2 infected hamsters treated with the anti-SARS-CoV-2 neutralizing monoclonal antibody (GenScript, 6D11F2) (30 mg/kg). Data are presented as mean ± SD. The statistical significances are marked on the graphs as *p < 0.05, **p < 0.01 and ***p < 0.001.

Fever, resulting from an innate immune response, is a common characteristic of infectious diseases like COVID-19. As expected, the control group infected with SARS-CoV-2 (IC) exhibited typical COVID-19 symptoms, with a rapid onset of hypothermia following a high fever, leading to death (Figure 2(a–d)). In contrast, the SARS-CoV-2-infected hamsters treated with the high dose of hzVSF-v13 (30 mg/kg, AHD) showed a slight increase in body temperature, gradually recovering to normal levels. Although the body temperatures of ALD and REM increased during the experimental period, REM showed a more significant increase, indicating infection-associated inflammation in both groups. In contrast, the body temperature changes in the neutralizing antibody groups (NLD and NHD) resembled those of the IC group, suggesting limited therapeutic efficacy of the neutralizing antibody in treating COVID-19 in the hamsters. Overall, body temperature measurements suggest that hzVSF-v13 is effective in treating COVID-19, even more so than remdesivir and the neutralizing antibody.

Unlike body temperature, the body weights of hamsters in all treated groups (ALD, AHD, NLD, NHD, and REM) were consistently maintained within a normal range during the viral challenge, while the body weight of the IC group decreased to 12 ± 4.5% by 4 dpi (Figure 2(b)), indicating a therapeutic efficacy in all three therapeutic agents: anti-eVIM (hzVSF-v13), the neutralizing antibody, and remdesivir. Notably, ALD and AHD tended to perform better than NLD, NHD, and REM, although the difference was not significant. In line with these disease manifestations, both ALD and AHD exhibited better disease outcomes than NLD and NHD, with a 60% survival rate (Figure 2(c)). Interestingly, the survival rate of REM was the poorest, even worse than the IC group. Given that remdesivir improved disease manifestations, we suspect that the low survival rate of REM may be attributed to the drug’s toxic effects rather than a lack of therapeutic efficacy.

In addition to assessing disease manifestations through body temperatures, body weights, survival rates and infection rate (Figure 2), observation of other disease manifestations, such as appearance, activity, labored breathing, dyspnea, cough, hunched posture, ruffled fur, and shaking chills, confirmed that ALD and AHD-treated hamsters were much healthier than those in the NLD, NHD, and REM groups, underscoring the effectiveness of anti-eVIM (hzVSF-v13) in treating COVID-19, even more so than remdesivir and the neutralizing antibody.

### The anti-eVIM treatment inhibited the formation of blood clots in the COVID-19 Roborovski SH101 hamsters

**Figure 3.**
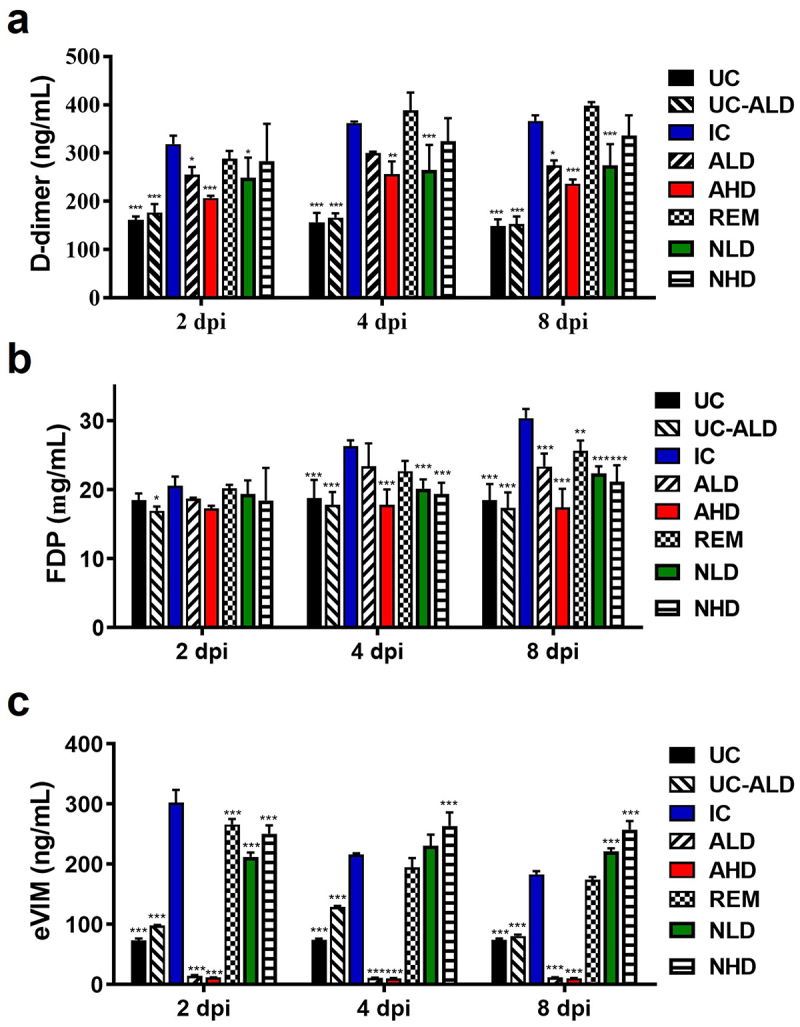
The anti-eVIM treatment inhibits the formation of blood clots in COVID-19 Roborovski SH101 hamsters. (a) the blood concentrations of D-dimer in the serum of Roborovski SH101 hamsters at 2, 4 and 8 dpi of SARS-CoV-2. (b) The blood concentrations of fibrinogen degradation products (FDP) in the serum of Roborovski SH 101 hamsters at 2, 4 and 8 dpi of SARS-CoV-2. (c) The blood concentrations of eVIM in the serum of Roborovski SH101 hamsters at 2, 4 and 8 dpi of SARS-CoV-2. UC, uninfection control; UC-ALD, uninfected hamster but injected with 10 mg/kg of hzVSF-v13; IC, infection control (the SARS-CoV-2-infected hamsters); ALD, the SARS-CoV-2-infected hamsters treated with low dose of anti-eVIM (hzVSF-v13 10 mg/kg); AHD, the SARS-CoV-2-infected hamsters treated with high dose of anti-eVIM (hzVSF-v13 30 mg/kg); REM, the SARS-CoV-2-infected hamsters treated with the therapeutic dose of remdesivir (5 mg/kg); NLD, the SARS-CoV-2-infected hamsters treated with the anti-SARS-CoV-2 neutralizing monoclonal antibody (GenScript, 6D11F2) (10 mg/kg); NHD, the SARS-CoV-2 infected hamsters treated with the anti-SARS-CoV-2 neutralizing monoclonal antibody (GenScript, 6D11F2) (30 mg/kg). Data are presented as mean ± SD. The statistical significances are marked on the graphs as *p < 0.05, **p < 0.01 and ***p < 0.001.

After observation of clinical symptoms, we started to analyze blood profile. The blood clot formation due to serious inflammation is one of the main characteristics of COVID-19, especially severe COVID-19. The blood levels of D-dimer and fibrin degradation products (FDP), the markers of blood clot formation, were measured to evaluate the formation of blood clots (Figure 3(a, b)). While the blood levels of both D-dimer and FDP were increased in the virus control, these blood markers of ALD and AHD were not increased or slightly increased within the range of a normal level (Figure 3(a, b)). Especially, the differences were most obvious at 4 dpi where the blood levels of both D-dimer and FDP of AHD were 29 ± 7% and 32 ± 5% lower than those of the virus control. The overall blood levels of D-dimer and FDP in AHD were indistinguishable to that of UC, indicating no blood clot formation in the treatment group. Unlike AHD, Remdesivir treatment (REM) did not inhibit the increasement of the blood concentrations of D-dimer and FDP, which means that remdesivir did not efficiently block the inflammation caused by SARS-CoV-2 infection. The neutralizing antibody treatment (NLD and NHD) also leads to increased blood concentrations of D-dimer and FDP although not as much as REM. These results indicate that the therapeutic efficacy of anti-eVIM (hzVSF-v13) is better than those of the neutralizing antibody and remdesivir, which is consistent with the above disease manifestation results (Figure 1)

The *i.v*. injection of anti-eVIM (hzVSF-v13) into the uninfected hamsters (UC-ALD) drastically reduced the blood concentrations of eVIM as expected (Figure 3(c)). While the blood concentrations of eVIM were 302.06 ± 21.32 ng/mL ~ 215.75 ± 2.46 ng/mL in the infection control group (IC), which was 2.90 ~ 4.12 times higher than that of the un-infection control group (UC). Unlike hzVSF-13, Remdesivir was not efficient in inhibiting the increase of the blood concentrations of eVIM, suggesting that remdesivir was not efficient as much as hzVSF-v13 in terms of COVID-19 treatment. SARS-CoV-2 neutralizing antibody treatment increased the blood concentrations of eVIM throughout the experimental period. The increase in eVIM in the SARS-CoV-2 neutralizing antibody treatment group (Figure 3(c)) is consistent with the poor therapeutic efficacy of the neutralizing antibody (Figure 2). The poor therapeutic efficacy of SARS-CoV-2 neutralizing antibodies is also consistent with previous studies [36].

### The anti-eVIM treatment suppressed inflammation in the COVID-19 Roborovski SH101 hamsters

**Figure 4.**
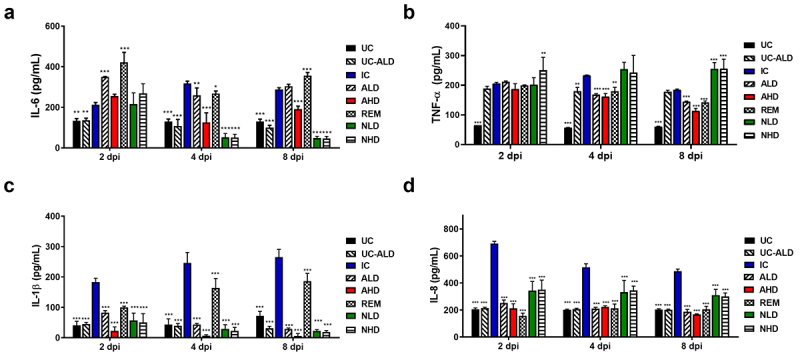
The anti-eVIM treatment inhibits generation of inflammatory cytokines in the blood of COVID-19 Roborovski SH101 hamsters. (a) the blood concentrations of IL-6 in the serum of COVID-19 Roborovski SH101 hamsters at 2, 4 and 8 dpi. (b) The blood concentrations of TNF-α in the serum of COVID-19 Roborovski SH101 hamsters at 2, 4 and 8 dpi. (c) The blood concentrations of IL-1β in the serum of COVID-19 Roborovski SH101 hamsters at 2, 4 and 8 dpi. (d) The blood concentrations of IL-8 in the serum of COVID-19 Roborovski SH101 hamsters at 2, 4 and 8 dpi. UC, uninfection control; UC-ALD, uninfected hamster but injected with 10 mg/kg of hzVSF-v13; IC, infection control (the SARS-CoV-2-infected hamsters); ALD, the SARS-CoV-2-infected hamsters treated with low dose of anti-eVIM (hzVSF-v13 10 mg/kg); AHD, the SARS-CoV-2-infected hamsters treated with high dose of anti-eVIM (hzVSF-v13 30 mg/kg); REM, the SARS-CoV-2-infected hamsters treated with the therapeutic dose of remdesivir (5 mg/kg); NLD, the SARS-CoV-2-infected hamsters treated with the anti-SARS-CoV-2 neutralizing monoclonal antibody (GenScript, 6D11F2) (10 mg/kg); NHD, the SARS-CoV-2 infected hamsters treated with the anti-SARS-CoV-2 neutralizing monoclonal antibody (GenScript, 6D11F2) (30 mg/kg). Data are presented as mean ± SD. The statistical significances are marked on the graphs as *p < 0.05, **p < 0.01 and ***p < 0.001.

Measurement of blood cytokines further validated the therapeutic benefit of hzVSF-v13 in the treatment of COVID-19. As shown in Figure 4(a), the blood concentration of IL-6 increased in all the three treatment groups: anti-eVIM (hzVSF-v13), the neutralizing antibody, and remdesivir. However, the increase of IL-6 was least in the high dose of anti-eVIM (AHD) compared to the rest of other groups. The blood concentrations of TNF-α were showed similar trends with those of IL-6; overall increases in all treatment groups but least in AHD (Figure 4(b)), meaning the least inflammation in AHD. In contrast to IL-6 and TNF-α, the blood concentrations of IL-1β, a central proinflammatory cytokine, maintained to a level of un-infection control at 2 dpi in both treatment groups of anti-eVIM and the neutralizing antibody. Interestingly, the blood IL-1β levels were even slightly decreased after 4 dpi in AHD (Figure 4(c)). Unlike anti-eVIM and the neutralizing antibody, the remdesivir treatment (REM) did not inhibit the increase of the blood IL-1β levels, indicating the limited ability in inhibiting inflammation. The blood concentrations of IL8 were not increased in the treatment groups of anti-eVIM and remdesivir, but slightly increased in the neutralizing antibody treatment groups (Figure 4(d)). Overall, the blood cytokine analysis indicates that anti-eVIM actively suppressed the inflammatory response in the body of the SARS-CoV-2-infected hamsters in a dose-dependent manner.

### Histological examination confirmed the absence of inflammation and mucus production in the lungs of the COVID-19 Roborovski SH101 hamsters after anti-eVIM treatment

**Figure 5.**
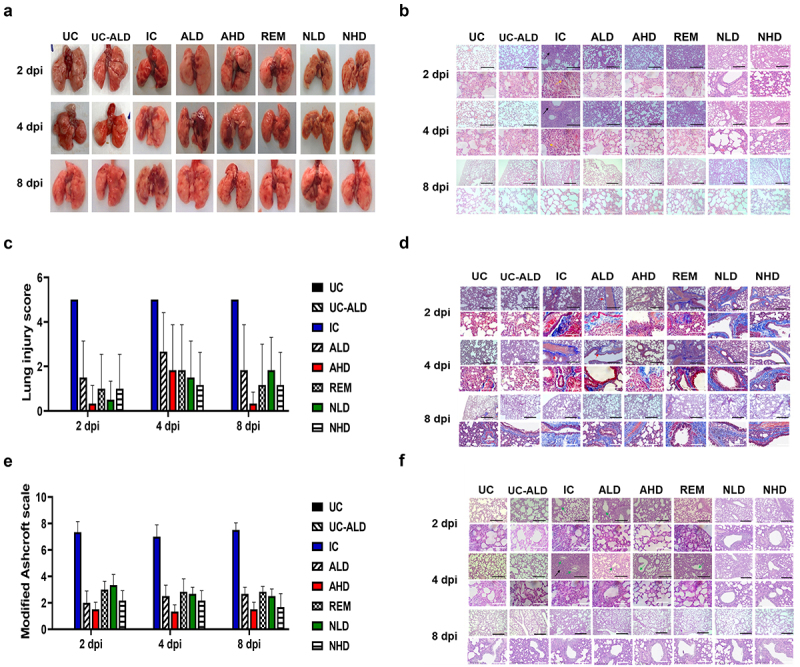
Histological examination shows that anti-eVIM treatment inhibits inflammation and mucus production in the lungs of COVID-19 Roborovski SH101 hamsters. (a) Photographic images of the dissected lungs of each Roborovski SH101 hamster group. (b) Representative images of H&E-stained lung sections from COVID-19 Roborovski SH101 hamsters at 2, 4, and 8 dpi, showing multifocal pneumonia with inflammatory cells (black arrows) and mononuclear cells (yellow arrow). (c) Lung injury scores. (d) Representative images of MT-stained lung sections from COVID-19 Roborovski SH101 hamsters at 2, 4, and 8 dpi. (e) Fibrosis scores. (f) Representative images of PAS-stained lung sections from COVID-19 Roborovski SH101 hamsters at 2, 4, and 8 dpi, indicating hyperplasia of goblet cells in alveolar septum (black arrow). UC, uninfection control; UC-ALD, uninfected hamster but injected with 10 mg/kg of hzVSF-v13; IC, infection control (the SARS-CoV-2-infected hamsters); ALD, the SARS-CoV-2-infected hamsters treated with low dose of anti-eVIM (hzVSF-v13 10 mg/kg); AHD, the SARS-CoV-2-infected hamsters treated with high dose of anti-eVIM (hzVSF-v13 30 mg/kg); REM, the SARS-CoV-2-infected hamsters treated with the therapeutic dose of remdesivir (5 mg/kg); NLD, the SARS-CoV-2-infected hamsters treated with the anti-SARS-CoV-2 neutralizing monoclonal antibody (GenScript, 6D11F2) (10 mg/kg); NHD, the SARS-CoV-2 infected hamsters treated with the anti-SARS-CoV-2 neutralizing monoclonal antibody (GenScript, 6D11F2) (30 mg/kg). The scale bars represent 100 μm for 100 × and 20 μm for 400 ×.

After completing clinical and serological analysis for each Roborovski SH101 hamster group, the lung tissue was excised and histologically observed. As shown in Figure 5(a), red spots were found in the lungs of the SARS-CoV-2 infected control group (IC) due to inflammation. It was shown that the pathological red spots were more abundant with a severe degree of infection, more severe at 2 dpi than at 4 dpi. As expected, the lungs of the three treatment groups – anti-eVIM (hzVSF-v13), the neutralizing antibody, and remdesivir – were not significantly different from those of the healthy control group. From this result, it is evident that overall lung inflammation was mild in all treatment groups due to their therapeutic efficacy.

The lungs isolated from each Roborovski SH101 hamster group were stained with H&E, MT and PAS, respectively (Figure 5(b–d)). According to the histological examination with H&E staining, it was confirmed that inflammatory cells significantly increased in the lung tissue of IC (Figure 5(b)). The lungs of AHD stained with H&E were not significantly different from the lungs of the healthy control group. However, slight inflammation was observed in ALD, REM, NLD, and NHD, although it was much milder than that of IC. MT staining demonstrated blue-stained tissues, which indicate fibrosis that occurs after inflammation due to SARS-CoV-2 infection (Figure 5(c)). Not surprisingly, extensive lung fibrosis was observed in the lungs of IC. The lungs of AHD were like those of UC, indicating a lack of fibrosis caused by inflammation. In the lungs of ALD, however, weakly stained blue tissue indicated the occurrence of lung fibrosis, although weak. However, in the lungs of REM, NLD, and NHD, there were obvious signs of fibrosis, as evident by blue-stained tissue areas. Finally, PAS staining was conducted to detect goblet cells that produce mucus due to SARS-CoV-2 virus infection (Figure 5(d)). It also showed that very few goblet cells were present in the lungs of AHD, ALD, and UC, indicating normal mucus production in AHD and ALD like UC. However, several goblet cells were observed in the lungs of REM, NLD, and NHD, indicating increased mucus production in the lungs of these hamsters.

### RT-PCR confirmed the therapeutic efficacy of anti-eVIM for the treatment of COVID-19 in the SARS-CoV-2-infected Roborovski SH101 hamsters

**Figure 6.**
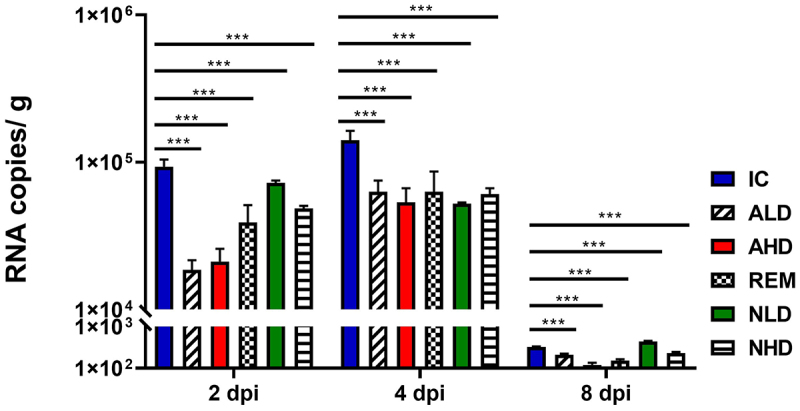
The viral replication in Roborovski SH101 hamsters infected with SARS-CoV-2. The viral RNA levels of SARS-CoV-2 in the lungs of Roborovski SH101 hamsters measured by RT-qPCR at 2, 4, and 8 dpi. IC, infection control (the SARS-CoV-2-infected hamsters); ALD, the SARS-CoV-2-infected hamsters treated with low dose of anti-eVIM (hzVSF-v13 10 mg/kg); AHD, the SARS-CoV-2-infected hamsters treated with high dose of anti-eVIM (hzVSF-v13 30 mg/kg); REM, the SARS-CoV-2-infected hamsters treated with the therapeutic dose of remdesivir (5 mg/kg); NLD, the SARS-CoV-2-infected hamsters treated with the anti-SARS-CoV-2 neutralizing monoclonal antibody (GenScript, 6D11F2) (10 mg/kg); NHD, the SARS-CoV-2 infected hamsters treated with the anti-SARS-CoV-2 neutralizing monoclonal antibody (GenScript, 6D11F2) (30 mg/kg). Data are presented as mean ± SD. The statistical significances are marked on the graphs as *p < 0.05, **p < 0.01 and ***p < 0.001.

As a final verification of antiviral efficacy, SARS-CoV-2 virus titers were quantitated by quantitating mRNA from the isolated lung tissue (Figure 6). The virus quantity in the IC group was 9.33 × 10^4^/g ±1.09 × 10^4^/g at 2 dpi and 1.41 × 10^5^/g ±2.21 × 10^4^/g at 4 dpi. In the ALD group, the viral titer was 1.86 × 10^4^/g ±2.97 × 10^3^/g at 2 dpi, representing an 80% decrease compared to that of the IC group. Similarly, in the AHD group, the viral titer was 2.13 × 10^4^/g ±14.69 × 10^3^/g at 2 dpi, showing a 77% decrease compared to that of the IC group. In the REM group, the viral titter was 3.91 × 10^4^/g ± 1.21 × 10^4^/g at 2 dpi, indicating a reduction to 41.9%. The viral titers of NLD and NHD were reduced to 5.23 × 10^4^/g ± 9.93 × 10^2^/g and 6.09 × 10^4^/g ± 5.84 × 10^3^/g, respectively, representing a 44.1% and 34.7% reduction compared to that of the IC group, respectively.

The relative virus quantities of each experimental group at 4 and 8 dpi remained consistent with those measured at 2 dpi. In the anti-eVIM treatment group, the viral quantity was lower than that in the neutralizing antibody and remdesivir treatment groups. The viral quantities in the neutralizing antibody and remdesivir treatment groups were fairly like each other. This virus quantification result indicates that the therapeutic efficacy of anti-eVIM was better than that of the neutralizing antibody and remdesivir, which is consistent with the results of the therapeutic evaluation above.

## Discussion

**Figure 7.**
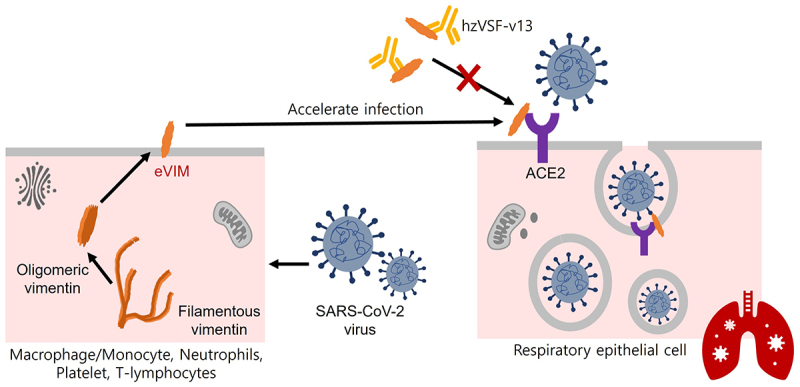
The working model explaining the antiviral role of hzVSF-v13. hzVSF-v13 is an anti-eVIM IgG4 monoclonal antibody that strongly binds to eVIM. This binding inhibits the catalytic activity of eVIM required for SARS-CoV-2 entry into its host cell, thereby impeding viral replication.

The eVIM has recently emerged as a key factor in the pathogenesis of viral infections, including SARS-CoV-2, by serving as a catalyst of viral entry into host cells [28,37]. Despite solid evidence, all these findings were based on *in vitro* experiments. In this work, we provide *in vivo* evidence for the first time that eVIM plays an important functional role in the pathogenesis of viral infection. Since eVIM plays a catalytic role facilitating the binding of a virus with its viral receptor (Figure 7), the propagation of viruses was correlated with the concentration of eVIM *in vitro* experiments [38,39]. Figure 1(b) shows that the propagation of SARS-CoV-2, as measured by viral titer, was well correlated with the blood concentration of eVIM in the experimental animal. Considering the ample presence of inherent interfering factors *in vivo*, the coefficient of determination in this work (Figure 1(b), R^2^ = 0.6394, *p* < 0.001) was strong enough to conclude that eVIM plays a key role of eVIM in the pathogenesis of viral infections.

Since eVIM plays a key role in the pathogenesis of viral infections in the Roborovski SH101 hamster, it is not surprising to find that removal of eVIM from the blood through intravenous treatment with anti-eVIM (hzVSF-v13) improved the clinical symptoms of the COVID-19 Roborovski SH101 hamsters. The COVID-19 Roborovski SH101 hamsters treated with intravenous injections of hzVSF-v13 did not show the typical clinical symptoms of COVID-19, such as snuffling, labored breathing, dyspnea, cough, hunched posture, progressive weight loss, ruffled fur, and high fever with shaking chills. In line with these subjective measures of clinical symptoms, the objective clinical indicators, including changes in body weight and body temperature, were dramatically improved in the hzVSF-v13 treatment groups in a dose-dependent manner (Figure 2a,b). The improvement of clinical symptoms with hzVSF-v13 injection was superior to that observed in the remdesivir or SARS-CoV-2 neutralizing antibody-treated groups.

COVID-19 can be categorized into mild and severe forms. In severe COVID-19 cases, SARS-CoV-2 infection is not limited to the respiratory tract but involves systemic infection, affecting organs, including the lungs [40–42]. Severe COVID-19 is characterized by inflammatory cytokine production and persistent inflammation in lung and other organs [43,44]. Excessive inflammation is the primary cause of morbidity and mortality in severe COVID-19 [45,46]. Therefore, anti-inflammation is a key therapeutic aim in the management of severe COVID-19 [47,48]. In this respect, the anti-inflammatory effect of hzVSF-v13 should be noted (Figure 4). Although hzVSF-v13 reduced pro-inflammatory cytokines, it most significantly reduced the blood concentration of IL-1β, a key mediator of the inflammatory response (Figure 4(c)), indicating excellent anti-inflammatory efficacy of hzVSF-v13. The therapeutic beneficial of hzVSF-v13 extends beyond its anti-inflammatory effect; it also inhibits SARS-CoV-2 propagation in the body by interfering the catalytic reaction of eVIM (Figure 1).

Following the emergence of COVID-19, hzVSF-v13 was administered to COVID-19 patients without animal studies in the initial emergency when no standard therapy was established [49–51]. Both investigator-initiated trials showed that hzVSF-v13 has excellent therapeutic efficacy for COVID-19, although the sample sizes were small, and experimental data were limited. This work, combined with previous small-scale clinical observations, provides a solid basis for stepwise clinical trials to develop hzVSF-v13 as a therapeutic agent for severe COVID-19.

Overall, the therapeutic efficacy of hzVSF-v13 for COVID-19 surpassed that of remdesivir or the SARS-CoV-2 neutralizing antibody in terms of improving clinical symptoms, histological examination, blood profiles, and viral titers. Considering that current therapeutic agents, such as neutralizing antibodies, molnupiravir, and nirmatrelvir/ritonavir, are primarily used for prevention of progression to severe COVID-19, and remdesivir is the only antiviral drug for severe COVID-19 [52–54], this work supports the development of hzVSF-v13 as a therapeutic agent for severe COVID-19. Additionally, this work provides an *in vivo* foundation for a possible development of hzVSF-v13 as a general antiviral drug for the treatment of various viral infections, such as SARS-CoV, cowpea mosaic virus, Japanese encephalitis virus, dengue virus, and human papillomavirus, in which eVIM plays an important pathogenic role [55–57].

## Conclusion

In conclusion, this study offers the first *in vivo* evidence of eVIM’s critical pathogenic role during viral infections. The use of an anti-eVIM monoclonal antibody to remove eVIM provided robust protection against SARS-CoV-2 in Roborovski SH101 hamsters. Given eVIM’s pivotal role in facilitating viral entry into host cells, its removal from the bloodstream led to the effective cure of SARS-CoV-2 infection in these animals. Notably, the therapeutic efficacy of hzVSF-v13 in treating COVID-19 surpassed that of both Remdesivir and a SARS-CoV-2 neutralizing antibody. Although this study focused solely on SARS-CoV-2, the cooperative role of eVIM with viral receptors in viral entry suggests that these findings could support the development of hzVSF-v13 as a broad-spectrum antiviral drug, offering potential treatment options for various viral infections beyond COVID-19.

## Supplementary Material

S_Fig1.jpg

S_Fig2.jpg

QVIR-2024-0683.R1-Supplementary files with legends.docx

S1Table.docx

## Data Availability

The data supporting the findings of this study are openly available in “figshare” at https://doi.org/10.6084/m9.figshare.27321297
