# Supplementary figures and images for: Reduction of extracellular vimentin in blood provides protection against SARS-CoV-2 infection
Source: Virulence. 2025 Oct 7;16(1):2568052. doi: 10.1080/21505594.2025.2568052 (PMC12505511; doi:10.1080/21505594.2025.2568052)

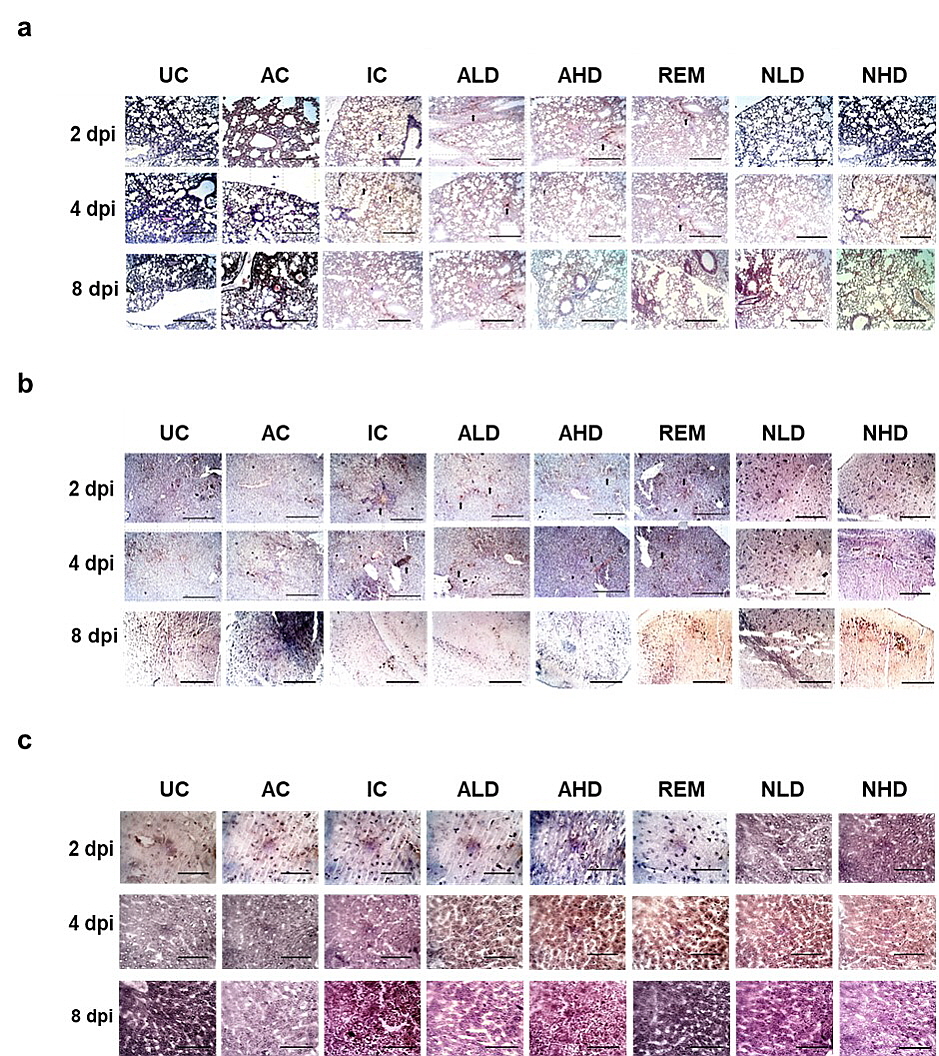

Supplement: S_Fig1.jpg [file KVIR_A_2568052_SM9156.jpg]

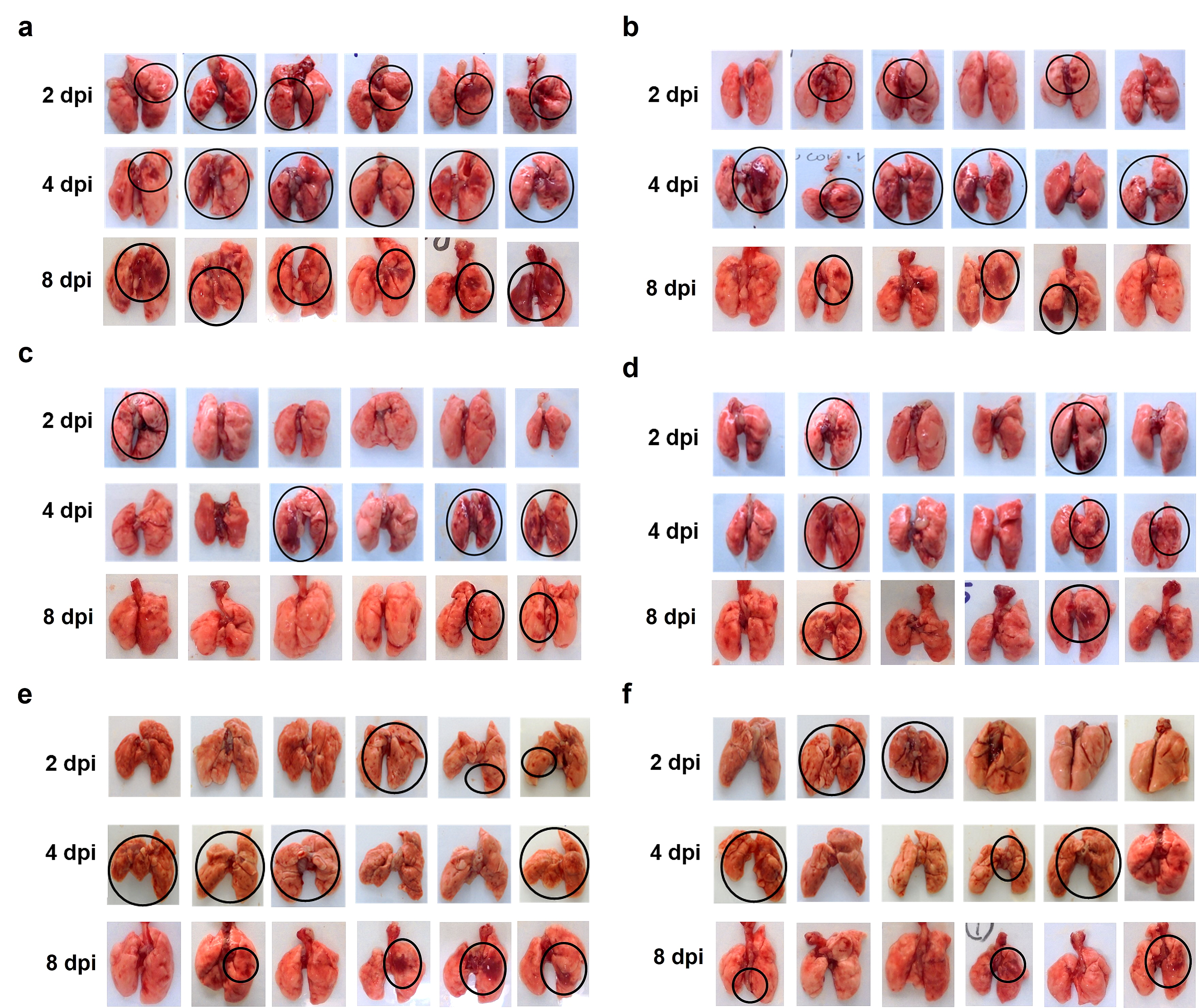

Supplement: S_Fig2.jpg [file KVIR_A_2568052_SM9155.jpg]
