# Supplementary material for: Reduction of extracellular vimentin in blood provides protection against SARS-CoV-2 infection
Source: Virulence. 2025 Oct 7;16(1):2568052. doi: 10.1080/21505594.2025.2568052 (PMC12505511; doi:10.1080/21505594.2025.2568052)
Supplement: QVIR-2024-0683.R1-Supplementary files with legends.docx [file KVIR_A_2568052_SM9154.docx]

**Supplementary information**

**
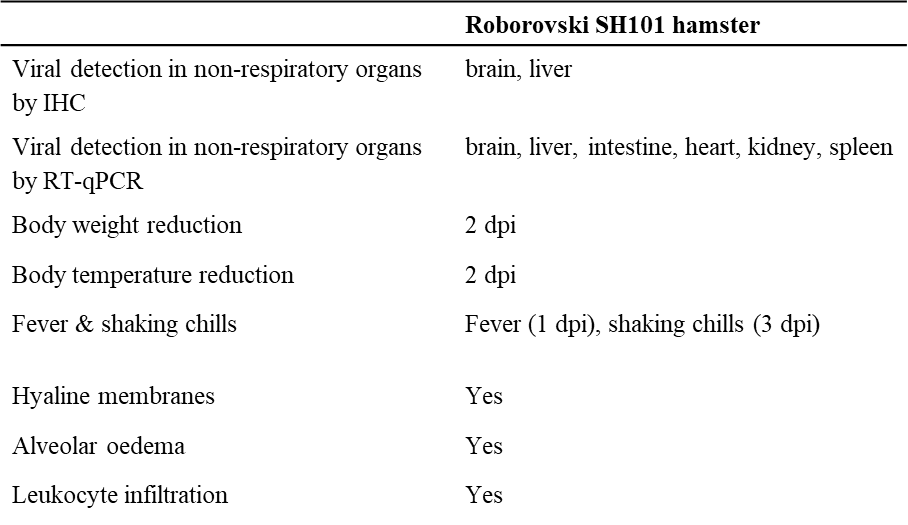
Supplementary Table 1. Summary of Disease Manifestations and Histology in the SARS-CoV-2 Infected Roborovski SH101 Hamster**

**
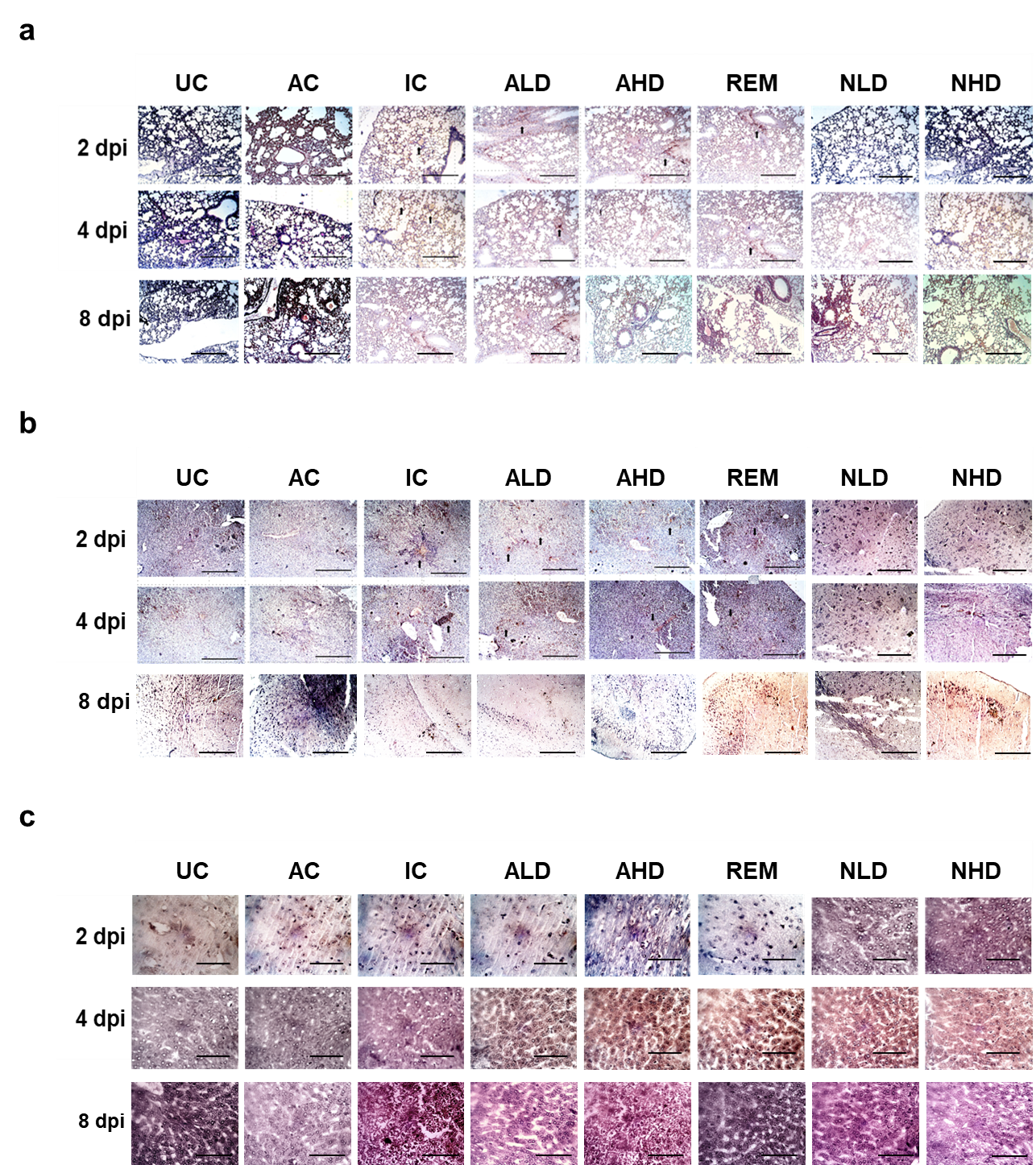
**

**Supplementary Figure 1. The Histological examination results of the SARS-CoV-2-infected Roborovski SH101 hamsters.** The representative images of the immunohistochemistry (IHC) of the (a) lung, (b) brain, and (c) liver of the Roborovski SH101 hamster at 2, 4, and 8 dpi. It has been observed that the brown portion of tissue increases according to the degree of viral infection (black arrow). UC, Uninfection control; UC-ALD, Uninfected hamster but injected with 10 mg/kg of hzVSF-v13; IC, Infection control (the SARS-CoV-2-infected hamsters); ALD, the SARS-CoV-2-infected hamsters treated with low dose of anti-eVIM (hzVSF-v13 10 mg/kg); AHD, the SARS-CoV-2-infected hamsters treated with high dose of anti-eVIM (hzVSF-v13 30 mg/kg); REM, the SARS-CoV-2-infected hamsters treated with the therapeutic dose of remdesivir (5 mg/kg); NLD, the SARS-CoV-2-infected hamsters treated with the anti-SARS-CoV-2 neutralizing monoclonal antibody (GenScript, 6D11F2) (10 mg/kg); NHD, the SARS-CoV-2 infected hamsters treated with the anti-SARS-CoV-2 neutralizing monoclonal antibody (GenScript, 6D11F2) (30 mg/kg). The scale bars represent 100 μm for 100ⅹ.

**
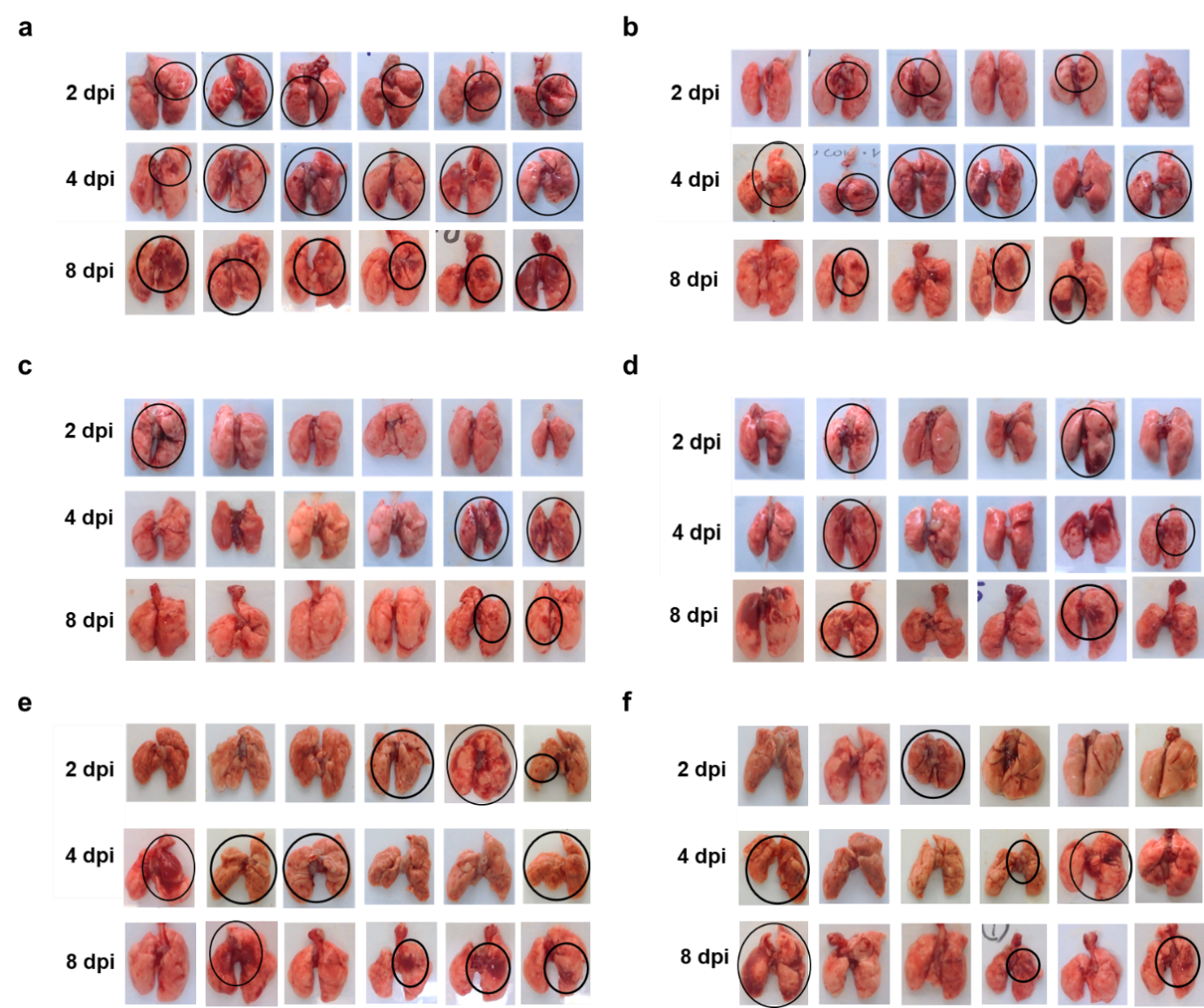
**

**Supplementary Figure 2. Fig.** **The photographic images of the dissected lungs of the SARS-CoV-2-infected Rovorovski SH101 hamster.** (a) IC, Infection control (the SARS-CoV-2-infected hamsters). (b) ALD, the SARS-CoV-2-infected hamsters treated with low dose of anti-eVIM (hzVSF-v13 10 mg/kg). (c) AHD, the SARS-CoV-2-infected hamsters treated with high dose of anti-eVIM (hzVSF-v13 30 mg/kg). (d) REM, the SARS-CoV-2-infected hamsters treated with the therapeutic dose of remdesivir (5 mg/kg). (e) NLD, the SARS-CoV-2-infected hamsters treated with the anti-SARS-CoV-2 neutralizing monoclonal antibody (GenScript, 6D11F2) (10 mg/kg). (f) NHD, the SARS-CoV-2 infected hamsters treated with the anti-SARS-CoV-2 neutralizing monoclonal antibody (GenScript, 6D11F2) (30 mg/kg).
