# Supplementary material for: Reduction of extracellular vimentin in blood provides protection against SARS-CoV-2 infection
Source: Virulence. 2025 Oct 7;16(1):2568052. doi: 10.1080/21505594.2025.2568052 (PMC12505511; doi:10.1080/21505594.2025.2568052)
Supplement: S1Table.docx [file KVIR_A_2568052_SM9153.docx]

**S1. Table. Summary of Disease Manifestations and Histology in the SARS-CoV-2 Infected Roborovski SH101 Hamster.**

|  | **Roborovski SH101 hamster** |
| --- | --- |
| Viral load in lung | 1.65E+06 |
| Viral load in trachea | 2.25E+06 |
| Viral detection in non-respiratory organs by IHC | Brain, liver, intestine |
| Viral detection in non-respiratory organs by RT-qPCR | Brain, liver, intestine, heart, kidney, spleen |
| Body weight reduction | 2 dpi |
| Body temperature reduction | 2 dpi |
| Fever & shaking chills | Fever(1 dpi), shaking chills (3 dpi) |
| Affected lung | 69 % |
| Hyaline membrane | Yes |
| Alveolar oedema | Yes |
| Leukocyte infiltration | Yes |
| Survival | 17 % |
